# Supplementary material for: In Situ Gene Expression in Native Cryofixed Bone Tissue
Source: Biomedicines. 2022 Feb 18;10(2):484. doi: 10.3390/biomedicines10020484 (PMC8962289; doi:10.3390/biomedicines10020484)
Supplement: Supplementary file 1 [file biomedicines-10-00484-s001.zip › Figure S1.pdf]

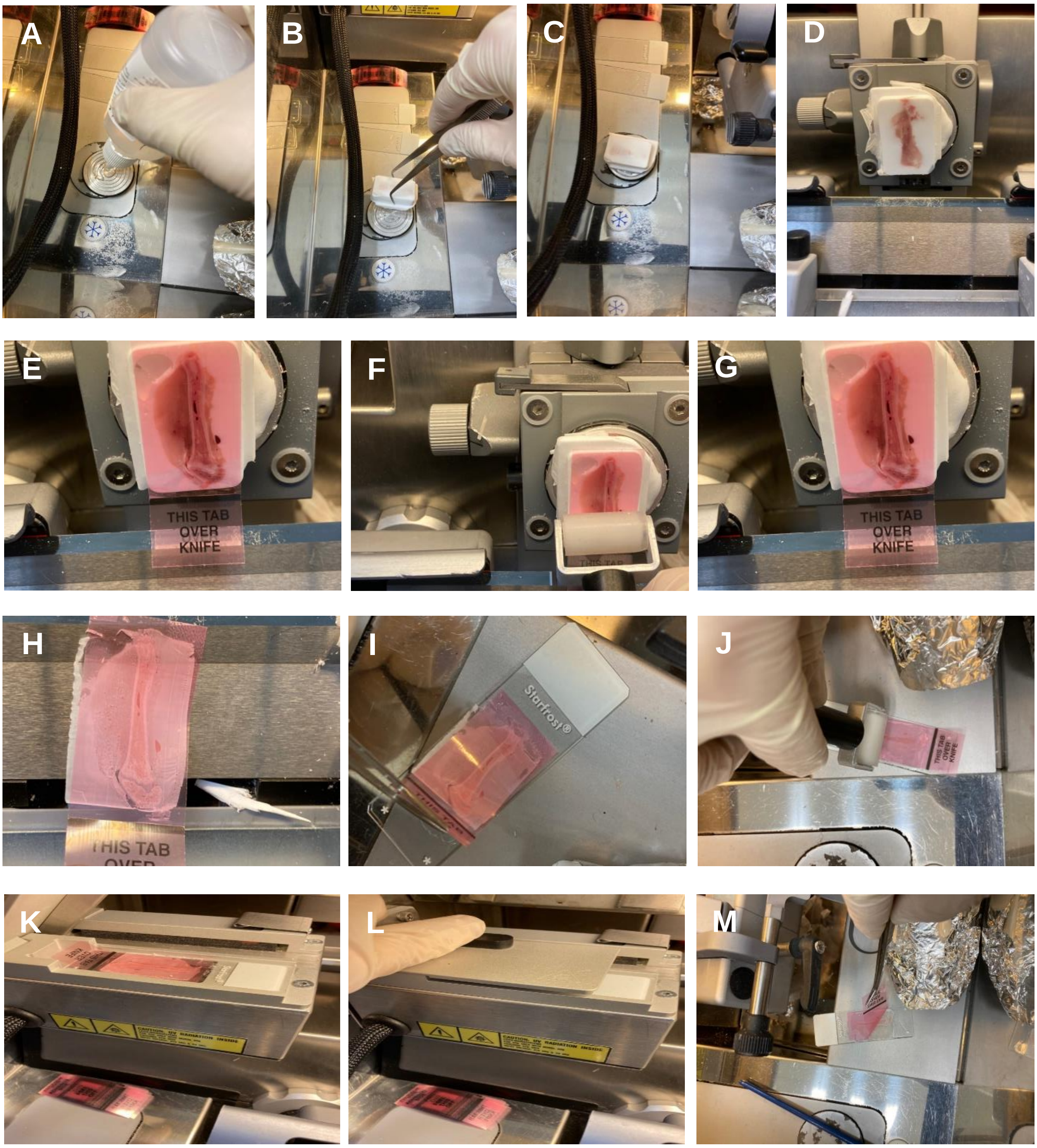

Figure S1. Workflow of cryo-sample preparation. (**A**) Covering mandrel with cryomount solution. (**B**) Positioning the block on the mandrel. (**C**) Hardening of the cryomount and fixation of the sample on the mandrel. (**D**) Trimming of the sample. (**E**) Positioning of the adhesive film on the surface of the block. (**F**) Application of the roller to improve the adhesion of the film. (**G**) Cutting the sample slowly and uninterruptedly by holding the lower part of the adhesive film. (**H**) Obtaining of 5 µm tissue section on adhesive film. (**I**) Positioning the adhesive film on the precoated slide. (**J**) Application of the roller to improve the adhesion to the slide. (**K**) Positioning the slide in CryoJane flash unit. (**L**) Triggering two flashes at 30 seconds intervals. (**M**) Removing the film with cold tool.
