# Supplementary material for: In Situ Gene Expression in Native Cryofixed Bone Tissue
Source: Biomedicines. 2022 Feb 18;10(2):484. doi: 10.3390/biomedicines10020484 (PMC8962289; doi:10.3390/biomedicines10020484)
Supplement: Supplementary file 1 [file biomedicines-10-00484-s001.zip › Figure S2.pdf]

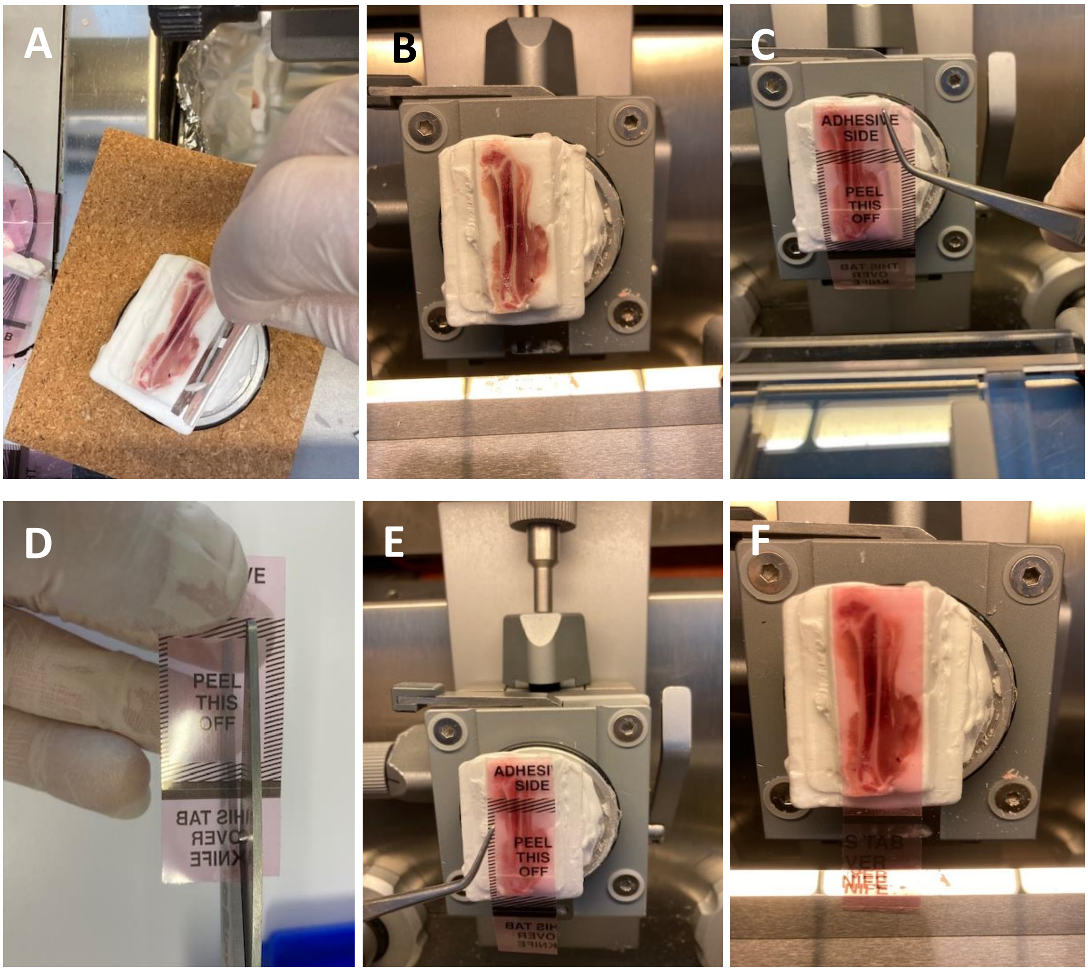

Figure S2. Workflow to optimize the transfer of the cryo-section. (**A**) Block trimming to adjust to the sample surface. (**B**) Positioning the block on the mandrel. (**C**) Estimation of the adhesive film surface. (**D**) Cutting of the adhesive film. (**E**) Positioning of the adhesive film on the surface of the block. (**F**) Adhesion of the film on the surface of the sample.
