# Supplementary material for: In Situ Gene Expression in Native Cryofixed Bone Tissue
Source: Biomedicines. 2022 Feb 18;10(2):484. doi: 10.3390/biomedicines10020484 (PMC8962289; doi:10.3390/biomedicines10020484)
Supplement: Supplementary file 1 [file biomedicines-10-00484-s001.zip › Table S1.pdf]

A

Rat (Ratus norvegicus) β-actin cDNA sequence and (T3 / T7) promoters

```
5'-
GCTACTTGCTGAATTAACCCTCACTAAAGGGAccattgaacacggcattgtcaccaactgggacgata
tggagaagatttggcaccacactttctacaatgagctgcgtgtggcccctgaggagcaccctgtgctg
ctcaccgaggcccctctgaaccctaaggccaaccgtgaaaagatgacccagatcatgtttgagacctt
caacaccccagccatgtacgtagccatccaggctgtgttgtccctgtatgcctctggtcgtaccactg
gcattgtgatggactccggagacggggtcacccacactgtgcccatctatgagggttacgcgctccct
catgccatcctgcgtctggacctggctggccgggacctgacagactacctcatgaagatcctgaccga
gcgtggctacagcttcaccaccacagctgagagggaaatcgtgcgtgacattaaagagaagctgtgct
atgttgccctagacttcgagcaagagatggccactgccgcatcctcttcctccctggagaagagctat
gagctgcctgacggtcaggtcatcactatcggcaatgagcggttccgatgccccgaggctctcttcca
gccttccttcctgggtatggaatcctgtggcatccatgaaactacattcaattccatcatgaagtgtg
acgttgacatccgtaaagacctctatgccaacacagtgctgtctggtggcaccaccatgtacccaggc
attgctgacaggatgcagaaggagattactgccctggctcctagcaccatgaagatcaagatcattgc
tcctcctgagcgcaagtactctgtgtggattggtggctctatcctggcctcactgtccaccttccagc
agatgtggatcagcaagcaggagtacgatgagtccggcccctccatcgtgcaccgcaaatgcttctag
gcggactgttactgagctgcgtttCCCTATAGTGAGTCGTATTACATTCGACT-3'
```

B

Oligo name	Gene accession number	Oligo sequence
DIG_Rat_actin _FW_T3	NM_031144.3	TGAATTAACCCTCACTAAAGGGACCATTGAACACGGCATTGTC
DIG_Rat_actin _RV_T7	NM_031144.3	TGTAATACGACTCACTATAGGGCAAACGCAGCTCAGTAACAGTCC
$N_{(11)}$-T3_promoter_ primer		GCTACTTGCTGAATTAACCCTCACTAAAGGGA
$N_{(9)}$-T7_promoter_ primer		AGTCGAATGTAATACGACTCACTATAGGG
HCR_rat_actin _b_revcom_fra gment1	NM_031144.3	CTCACTCCCAATCTCTATCTACCCTACAAATCCAATAAAAAACCCAT ACCCACCATCACACCCTGGTGCCTAGGGCGGCCCACGATGGAGG ATTTTCACTTCATATCACTCACTCCCAATCTCTATCTACCC
HCR_rat_actin _b_revcom_fra gment2	NM_031144.3	CTCACTCCCAATCTCTATCTACCCTACAAATCCAATAAAAATTGAA GGTCTCAAACATGATCTGGGTCATCTTTTCACGGTTGGCCTTAGGA TTTTCACTTCATATCACTCACTCCCAATCTCTATCTACCC
HCR_rat_actin _b_revcom_fra gment3	NM_031144.3	CTCACTCCCAATCTCTATCTACCCTACAAATCCAATAAAAAGTTTCA TGGATGCCACAGGATTCCATACCCAGGAAGGAAGGCTGGAAGAG ATTTTCACTTCATATCACTCACTCCCAATCTCTATCTACCC
HCR_rat_actin _b_revcom_fra gment4	NM_031144.3	CTCACTCCCAATCTCTATCTACCCTACAAATCCAATAAAAAACTTGC GCTCAGGAGGAGCAATGATCTTGATCTTCATGGTGCTAGGAGCCA TTTTCACTTCATATCACTCACTCCCAATCTCTATCTACCC
HCR_rat_actin _b_revcom_fra gment5	NM_031144.3	CTCACTCCCAATCTCTATCTACCCTACAAATCCAATAAAAAATCGTA CTCCTGCTTGCTGATCCACATCTGCTGGAAGGTGGACAGTGAGGA TTTTCACTTCATATCACTCACTCCCAATCTCTATCTACCC

Table S1. Schematic representation of the β-actin *in vitro* transcription. (**A**) Rat β-actin cDNA sequence (944bp) in black linked to sequences (in red) containing T3 and T7 promoters in 5' and 3', respectively (framed). (**B**) Oligos used for *in situ* hybridization experiments. T3 (T3 promoter), T7 (T7 promoter), FW (forward primer), RV (reverse primer).
